# Ampullary cancer detected upon re‐examination in a patient initially diagnosed as cancer of unknown primary

**DOI:** 10.1002/jgh3.12710

**Published:** 2022-02-08

**Authors:** Yasuharu Kawamoto, Kenji Ikezawa, Shinichiro Hasegawa, Hiroshi Wada, Toshihiro Kudo, Shigenori Nagata, Kazuyoshi Ohkawa

**Affiliations:** ^1^ Department of Hepatobiliary and Pancreatic Oncology Osaka International Cancer Institute Osaka Japan; ^2^ Department of Gastroenterological Surgery Osaka International Cancer Institute Osaka Japan; ^3^ Department of Medical Oncology Osaka International Cancer Institute Osaka Japan; ^4^ Department of Diagnostic Pathology and Cytology Osaka International Cancer Institute Osaka Japan

**Keywords:** cancer of unknown primary site, conversion surgery, duodenal papillary carcinoma, endoscopic ultrasound

## Abstract

In patients with cancer of unknown primary (CUP), the efficiency of reexamination in the improvement of the prognosis has not been demonstrated yet. In the present case, ampullary adenocarcinoma, initially diagnosed as CUP, was revealed by endoscopic forceps biopsy for the ampullary lesion progressing over time. Reexamination of the primary site in patients with CUP could contribute to better treatment options and improvement in the prognosis.

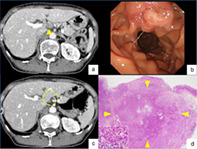

A woman in her 60s with fatigue and weight loss was referred to our hospital because of increased fluorodeoxyglucose uptake detected in the pancreatic head upon positron emission tomography‐computed tomography (PET/CT) scan. Laboratory data revealed no abnormality, including tumor markers. Contrast‐enhanced CT revealed an 18‐mm enlarged lymph node, adjacent to the pancreatic head (Fig. 1a), while no mass was detected in the head of the pancreas. Endoscopic examination with a side‐viewing duodenoscope revealed no apparent abnormality in the papilla of Vater (Fig. 1b). Although abnormal blood vessels were suspected under observation with narrow‐band imaging, no tumor was detected by forceps biopsy. Since no space‐occupying lesion was detected with endoscopic ultrasound (EUS) using the water immersion technique, ampullary cancer of the nonexposed protruded‐type was unlikely at that point. EUS‐guided fine needle aspiration (FNA) for the enlarged lymph node revealed poorly differentiated adenocarcinoma (Fig. 1c,d). Since immunohistochemical examinations did not indicate the primary site of the cancer, the patient was diagnosed with cancer of unknown primary (CUP). Following six courses of chemotherapy with carboplatin and paclitaxel, the appearance of multiple lymph node metastases was observed (Fig. 2a). Although we aimed to perform EUS‐FNA again for obtaining the tissues for comprehensive genomic profiling, endoscopic observation revealed ulcerated tumor in the papilla of Vater (Fig. 2b). Poorly differentiated adenocarcinoma was diagnosed with immunohistochemical analyses following forceps biopsy of the lesion. After the diagnosis of ampullary cancer, the patient underwent combination chemotherapy with gemcitabine, cisplatin, and S‐1, which resulted in a partial response with remarkable shrinkage of the enlarged lymph nodes (Fig. 2c). The patient underwent pancreaticoduodenectomy with lymph‐node dissection, which led to the final diagnosis of Stage IIIB (ypT2N2M0) ampullary adenocarcinoma (Fig. 2d). The patient did not show recurrence 8 months following the surgery.

**Figure 1:**
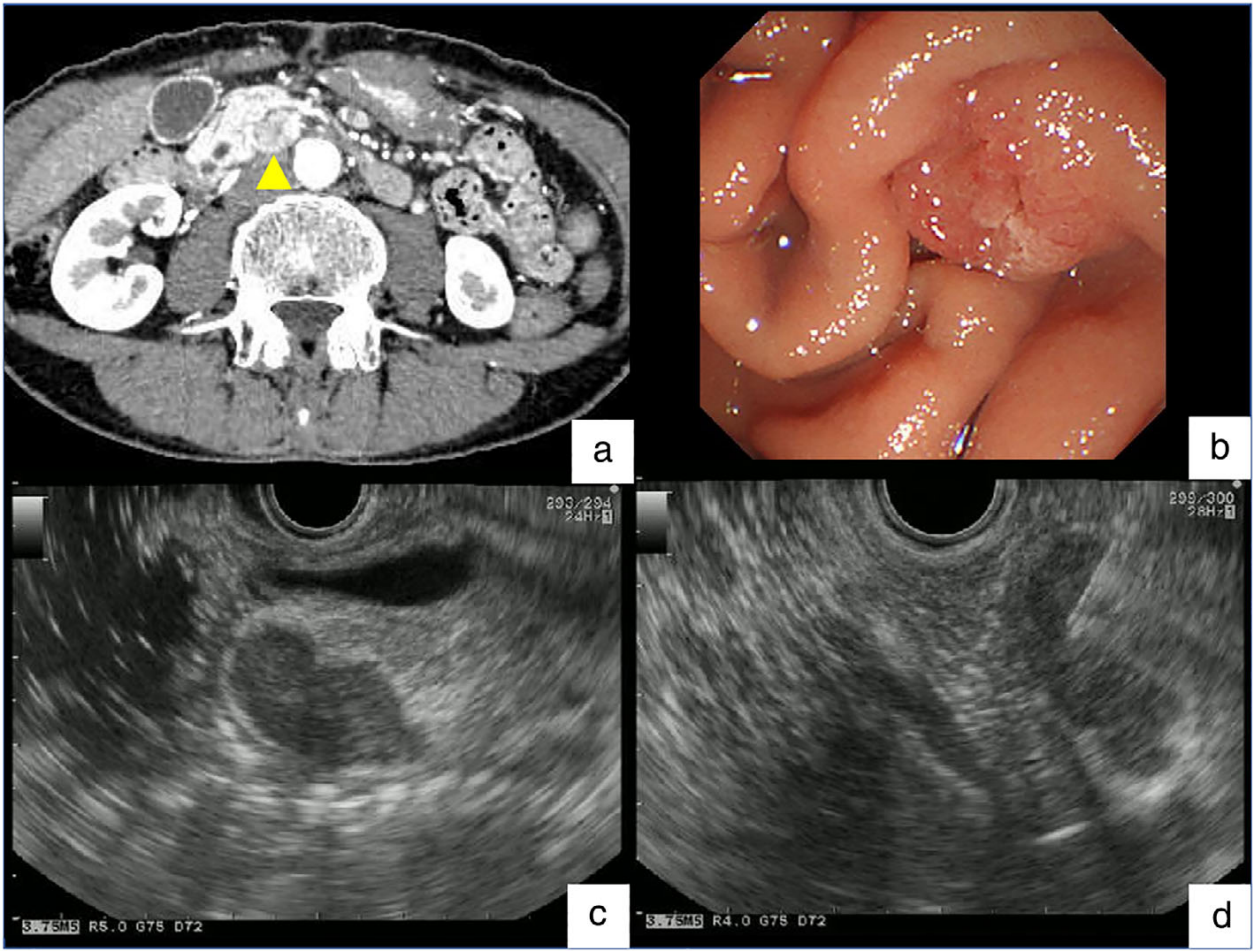
(a) Contrast‐enhanced computed tomography (CT) showing an 18‐mm enlarged lymph node (LN), adjacent to the pancreatic head (arrowhead). (b) Side‐viewing duodenoscopy, revealing no apparent abnormality on the papilla of Vater. (c) Observation with endoscopic ultrasound (EUS) using water immersion technique, revealing a hypoechoic mass with irregular contour (enlarged LN), which ruled out ampullary cancer of the nonexposed protruded‐type. (d) EUS‐guided fine needle aspiration with 22‐gauge needle for the enlarged LN.

**Figure 2:**
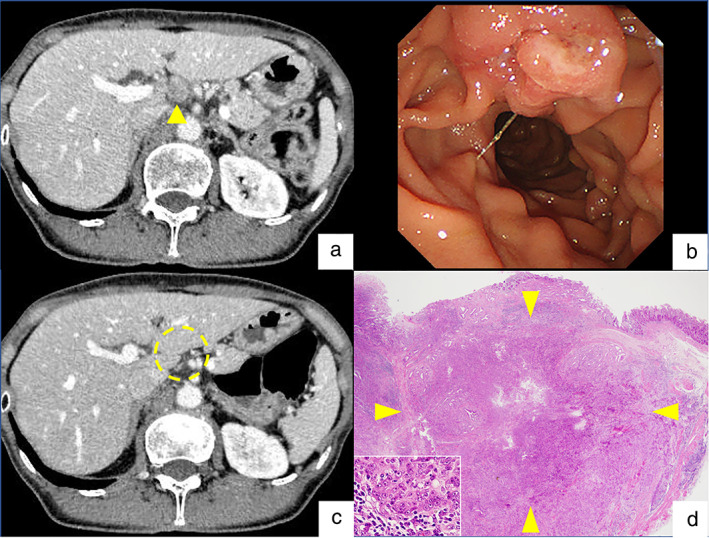
(a) Appearance of hilar lymph node (LN) enlargement following chemotherapy with carboplatin and paclitaxel (arrowhead). (b) Ulcerated tumor on the papilla of Vater under the observation with side‐viewing endoscopy, which was pathologically diagnosed as ampullary adenocarcinoma. (c) Disappearance of the hilar LN enlargement following combination chemotherapy with gemcitabine, cisplatin, and S‐1 (yellow circle). (d) Photomicrograph of stage‐IIIB (ypT2N2M0) primary ampullary carcinoma (arrowheads) in the resected specimen (H&E stain, ×20) with a small window of higher magnification in left lower corner (H&E, ×240).

CUP is pathologically diagnosed as metastatic carcinoma in which the obvious anatomical primary site is not identified after adequate diagnostic work‐up and occupies 2–5% of all diagnosed cancers.^1^, ^2^ In cases where re‐examination of the primary organ is performed, the identification rate of the primary site is as low as 7.6% in the patients with CUP.^3^ The guidelines for CUP published in Japan do not recommend routine re‐examination of the primary organ since the efficiency of re‐examination in the improvement of the prognosis has not been demonstrated yet.^4^ Although previous studies have reported that re‐examination of the primary organ in patients with CUP could identify various primary sites including the lung, breast, stomach, and colon,^5^, ^6^, ^7^ there have been no reports wherein ampullary cancer was identified as the primary site of CUP. Ampullary cancers are rare cancers that account for 0.2% of all gastrointestinal cancers and primarily manifest as obstructive jaundice.^8^, ^9^, ^10^ In the present case, ampullary adenocarcinoma, initially diagnosed as CUP, was revealed by endoscopic forceps biopsy for the ampullary lesion progressing over time. Re‐examination of the primary site in patients with CUP could contribute to better treatment options and improvement in the prognosis, highlighting the importance of re‐examination of the primary organs.

## Patient consent statement

Written informed consent was obtained from the patient for the publication of this case report and accompanying images.
